# Editorial: The infection and immunity of sexually transmitted disease pathogens

**DOI:** 10.3389/fcimb.2024.1498441

**Published:** 2024-10-17

**Authors:** Shuping Hou

**Affiliations:** Department of Dermatology and Venereology, Tianjin Medical University General Hospital, Tianjin, China

**Keywords:** HIV, Chlamydia trachomatis, infection, immunity, drug resistance

Sexually transmitted diseases (STDs) are a category of infections transmitted primarily through sexual contact, which pose significant risks to both physical and mental health and represent a critical concern for global public health (Gottlieb et al., 2024).The treatment and prevention of sexually transmitted diseases (STDs) continue to face significant challenges. This Research Topic aims to investigate the infection and immune mechanisms of sexually transmitted disease (STD) pathogens, with particular emphasis on their strategies for survival and dissemination within hosts, as well as the mechanisms of immune evasion and drug resistance. The insights obtained from these researches could provide valuable theoretical support for developing more effective treatment and prevention strategies in the future.

Chlamydia trachomatis (CT) is one of the most prevalent bacterial sexually transmitted infections worldwide, with an estimated 131 million new cases annually (Newman et al., 2015). While CT infections are frequently asymptomatic, they can lead to serious reproductive health issues, including female tubal infertility. Despite ongoing control programs, the prevalence of CT infections remains high. In the U.S., the incidence rates of reported CT infections increased steadily between 2000 and 2019 (Bedno et al., 2023).

Therefore, developing an effective Chlamydia trachomatis (CT) vaccine is a global health priority to reduce its infection rate and associated disease burden. A key aspect of this development involves identifying factors associated with protection against CT in humans, which include both immune markers and clinical outcomes. Yu et al. evaluated the correlations between phagocytosis activity and IgG1 responses targeting Chlamydia trachomatis (CT) elementary bodies (EBs) and antigens. They found that IgG1 ELISA, especially the Pgp3 IgG1 ELISA, was more sensitive than traditional total IgG ELISA for assessing CT antibody responses. Despite 95% of women with CT infection showing antibody-mediated phagocytosis of CT EBs, neither the detection of CT IgG1 antibodies nor antibody-mediated phagocytosis served as reliable correlates of protection against CT infection in humans. To aid in vaccine development, future research should explore alternative pathways through which antibodies confer immune protection.

The human immunodeficiency virus (HIV) is a Lentivirus within the retroviridae family and is known to cause acquired immune deficiency syndrome (AIDS). 39.9 million people globally were living with HIV in 2023 (UNAIDS, 2023). Sub-Saharan Africa bears the highest prevalence of HIV infection, representing over 70% of the global burden (Kharsany and Karim, 2016).

Significant advances have been made in understanding the molecular interactions between HIV and host cells, the host cell responses to the virus, and the potential therapeutic implications of these interactions (Jones et al., 2019; Shcherbatova et al., 2020). However, despite these advancements, a cure for HIV infection has remained elusive for over four decades (Shcherbatova et al., 2020; Sankaranantham, 2019). There are still many aspects of the virus-host cell interaction mechanisms that are not fully understood, and further exploration of these areas may be crucial for developing novel therapeutic strategies aimed at curing HIV infection (Shcherbatova et al., 2020; Bailon et al., 2020).

The development of antiretroviral drugs (ARVs) represents a significant landmark in the management of HIV infection. These drugs function by inhibiting viral activity within host cells, thereby reducing cellular damage and extending viral latency. However, the quest for an effective HIV vaccine remains fraught with challenges, primarily due to the virus’s genetic diversity and its complex mechanisms of immune evasion. In this context, Haynes et al. (2023) proposed a novel vaccine strategy involving a multi-stage immunogen design. This approach aims to sequentially activate and expand rare broadly neutralizing antibody (bnAb) B cell lineages, with the goal of creating immunogens that selectively induce critical antibody mutations.

Zhang et al. proposed and validated the “pathological proliferation” hypothesis for immune non-responders (INRs), indicating that in HIV-infected INRs, CD4+ T cells underwent excessive proliferation linked to abnormal activation, aging, and immune dysfunction. This over-proliferation of poor-quality cells resulted in incomplete recovery of CD4+ T cell count and function. Consequently, interventions aimed at enhancing CD4+ T cell proliferation or function in INRs may offer therapeutic benefits. This research provides new insights and potential intervention targets for improving immune reconstruction in individuals with HIV infection.

Anal intercourse significantly increases the risk of HIV in men who have sex with men (MSM), while oral sex can introduce gut-derived microbes into the saliva, potentially disrupting oral microecology in individuals with HIV. Despite its implications, this issue has been understudied. Guo et al. investigated the effects of HIV positivity on the KEGG functions and metabolic pathways of saliva bacteria in MSM. Their study revealed that as HIV progresses, anal sex notably alters the bacterial functions and metabolic pathways in MSM saliva. This alteration may be linked to HIV-induced changes in energy metabolism and heightened pathogen virulence. Particularly during acute AIDS and immune cell destruction stages, increased infection and resistance could indicate elevated risks of systemic and oral diseases in MSM individuals.

Giammarino et al. evaluated the synergistic antiretroviral activity of the naturally occurring dipeptide tryptophan-glycine (WG) in combination with existing antiretroviral drugs against multi-drug-resistant HIV-1 strains. Their findings demonstrated that WG-am exhibited moderate to strong synergy with the four tested antiretrovirals: raltegravir, tenofovir, efavirenz, and darunavir. This suggests that WG, which is enhanced in elite controllers, could emerge as a potential therapeutic option for treating HIV-1 strains resistant to these major antiretroviral compounds.

The surgical management of HIV/AIDS patients is distinct, with the risk of occupational exposure during the procedure primarily influenced by the patient’s HIV viral load. To standardize perioperative antiretroviral therapy (ART) for these patients, Ma et al. reported an expert consensus developed by the Surgery Group of the Chinese Association of STD and AIDS Prevention and Control, in collaboration with the Treatment Association and the Surgery Group of the Chinese Medical Association of Tropical Diseases and Parasitology. This consensus addresses various aspects, including surgical risk assessment, ART regimen selection, and prevention of opportunistic infections, with a particular emphasis on achieving rapid viral load reduction and immune function restoration before surgery.

In summary, this topic reveals the intricate biological mechanisms of STD pathogens and offers innovative solutions to address these challenges. By leveraging these research findings, we aim to reduce the global public health burden of STDs and encourage further research and clinical applications in related fields.
